# Lipid production from biofilms of *Marinobacter atlanticus* in a fixed bed bioreactor

**DOI:** 10.1186/s12934-024-02617-5

**Published:** 2024-12-19

**Authors:** Matthew D. Yates, Rebecca L. Mickol, Joseph S. Tolsma, Maryssa Beasley, Jamia Shepard, Sarah M. Glaven

**Affiliations:** 1 Center for Biomolecular Science and Engineering, US Naval Research Laboratory, Washington, DC 20375 USA; 2 Present Address: Catalent Pharma Solutions, Kansas City, MO 64137 USA

**Keywords:** Biofilm, Biomanufacturing, Lipids, Fixed-bed bioreactor, Additive manufacturing

## Abstract

**Background:**

Biotechnologies that utilize microorganisms as production hosts for lipid synthesis will enable an efficient and sustainable solution to produce lipids, decreasing reliance on traditional routes for production (either petrochemical or plant-derived) and supporting a circular bioeconomy. To realize this goal, continuous biomanufacturing processes must be developed to maximize productivity and minimize costs compared to traditional batch fermentation processes.

**Results:**

Here, we utilized biofilms of the marine bacterium, *Marinobacter atlanticus*, to produce wax esters from succinate (i.e., a non-sugar feedstock) to determine its potential to serve as a production chassis in a continuous flow, biofilm-based biomanufacturing process. To accomplish this, we evaluated growth as a function of protein concentration and wax ester production from *M. atlanticus* biofilms in a continuously operated 3-D printed fixed bed bioreactor. We determined that exposing *M. atlanticus* biofilms to alternating nitrogen-rich (1.8 mM NH_4_^+^) and nitrogen-poor (0 mM NH_4_^+^) conditions in the bioreactor resulted in wax ester production (26 ± 5 mg/L, normalized to reactor volume) at a similar concentration to what is observed from planktonic *M. atlanticus* cells grown in shake flasks previously in our lab (ca. 25 mg/L cell culture). The wax ester profile was predominated by multiple compounds with 32 carbon chain length (C_32_; 50–60% of the total). Biomass production in the reactor was positively correlated with dilution rate, as indicated by protein concentration (maximum of 1380 ± 110 mg/L at 0.4 min^−1^ dilution rate) and oxygen uptake rate (maximum of 4 mmol O_2_/L/h at 0.4 min^−1^ dilution rate) measurements at different flow rates. Further, we determined the baseline succinate consumption rate for *M. atlanticus* biofilms to be 0.16 ± 0.03 mmol/L/h, which indicated that oxygen is the limiting reactant in the process.

**Conclusion:**

The results presented here are the first step toward demonstrating that *M. atlanticus* biofilms can be used as the basis for development of a continuous flow wax ester biomanufacturing process from non-sugar feedstocks, which will further enable sustainable lipid production in a future circular bioeconomy

**Supplementary Information:**

The online version contains supplementary material available at 10.1186/s12934-024-02617-5.

## Introduction

Lipids are a critical class of molecules that are used in a wide range of products across many different commercial sectors, including chemicals, fuels, cosmetics, and pharmaceuticals [1–6]. Lipids used in these products are primarily derived from petroleum, but there has been increasing interest in establishing more sustainable sources of these materials [7], such as those produced by biology. The primary source of biologically-derived lipids is currently from plant material, such as the jojoba plant. However, plants with high lipid contents are found only in regions with specific climates and the yield per area of cultivation is low and competes for arable land with other industries, such as food and textiles [8]. In recent years, there has been increased interest in developing processes that utilize microorganisms for lipid production. Microorganisms, such as the marine, biofilm-forming bacterium, *Marinobacter atlanticus*, are able to naturally accumulate lipids derived from non-sugar feedstocks (such as organic acids and alkanes, which are potential next generation feedstocks [9]) as a response to environmental stress (*e.g.,* nitrogen limitation). Developing process(es) that incorporate these types of microorganisms is an interesting alternative for sustainable lipid production because they can: (i) convert a wide range of substrates as a feedstock for production [10], (ii) be scaled in an industrial process [11], (iii) produce a range of lipid products [12], and (iv) be genetically modified to potentially increase overall productivity and alter product profile [13]. The primary lipid product generated by *M. atlanticus* are wax esters, which encompass a large range of neutral lipids that consist of an ester of a fatty acid and fatty alcohol. Bacterial wax esters commonly have chain lengths in the range of C_28_ to C_36_ with varying lengths and degrees of unsaturation of the fatty acid and fatty alcohol fractions. For example, previous literature has shown that *Psychrobacter cryohalolentis* K5 produces primarily saturated C_36_ wax esters. In contrast, *Marinobacter aquaeolei* VT8 produces primarily C_34_ wax esters with a significantly higher proportion of unsaturation in the fatty acid and alcohol fractions [14], highlighting the complex nature of bacterial waxes that can be obtained depending on the production strain and substrate(s) used. These molecules, which are produced as a stress response to a nutrient limitation (primarily nitrogen), are critical to many applications, including lubricants, cosmetics, pharmaceuticals, and textile additives [15].

To date, lipid production from microorganisms has been explored in pilot-scale continuously-stirred tank reactors (CSTRs) primarily with planktonic microalgae and yeast cells to produce biodiesel precursors from sugar-based feedstocks [16]. These organisms have received considerable attention due to the high percentage of lipids within the cell mass (exceeding 80% in some cases) and the availability of robust genetic tools to modify these organisms. For example, lipid production in yeast has been tested at the pilot-scale in semi-continuous (250 L) [17] and batch (1000 L) [18] CSTRs to convert starch hydrolysate or pretreated corn stover to lipids with a range of titers from 5 to 16 g/L and productivities of 80 to 120 mg/L/h. Lipid accumulation in planktonic bacteria has also been tested in CSTRs at a similar scale and produced similar titer (ca. 6 g/L) and productivity (ca. 130 mg/L/h) [19] as microalgae and yeast. However, there are significantly fewer reports of using bacteria for lipid production. In all of the aforementioned previous reports, the production of lipids was performed in large, well-mixed, stainless steel tanks with planktonic cells to convert the sugar-based feedstock to a lipid product, which can consist of wax esters, triacylglycerides, and polyhydroxyalkanoates, among others [20].

While these efforts represent significant progress toward sustainable lipid production, a previous economic analysis indicated that the key challenge for cost-competitive production of microbial lipids is the development of a process that can continuously produce the lipid product [21]. Overcoming this hurdle requires development of novel manufacturing processes that are not based on traditional batch fermentations. Previous work has shown that production runtimes can be extended by using a fed-batch fermentation process, which more closely approximates a continuous production system, but still requires significant downtime compared to a truly continuous production system [22]. One alternative to a traditional batch or fed-batch fermentation process in a CSTR is to develop a continuous process in a fixed bed bioreactor utilizing cells that naturally attach to solid surfaces (*i.e*., a biofilm) or are synthetically confined to a surface [23, 24]. Fixed bed bioreactors function by maintaining the active cell layer on the surface of an internal support matrix with fluid that flows through the pores of the support material. The major advantage of a fixed bed bioreactor is that more active cells can inhabit the reactor (on a per volume basis), enabling higher production rates and increased efficiencies in some cases [25]. However, careful attention must be given to reactor conditions to prevent concentration gradients that decrease reaction rates [26]. Utilizing biofilms or immobilized cells in biotechnology is relatively common for wastewater treatment processes, such as trickling filters or granular activated sludge [27, 28], where the primary goal is removal of organic carbon from the water. However, biofilm-based processes for production of molecules with industrial relevance have been comparatively underexplored, in general, and particularly in the production of microbial lipids, primarily due to concerns with diffusion of substrates into or products out of the multi-cell layered film [29]. Much of the work to date on immobilized cells for bioproduction has focused on immobilizing cells in a synthetic matrix to increase the titers and yields of fermentative processes that produce soluble primary end products. For example, previous studies have shown that immobilization of *Saccharomyces cerevisiae* inside of a 3-D printed polymer matrix resulted in a 13% increase in ethanol yield from glucose [23]. Further, immobilization of *Propionibacterium acidipropionici* in a fibrous bed bioreactor resulted in a ca. 20% increase in propionic acid production [30] when operated in a batch fermentation. In other applications, cells engineered to express a desired enzyme have been embedded in a synthetic matrix at high cell densities to increase the reaction rate to a desired product. In one instance, *Escherichia coli* cells engineered to overexpress a carbonic anhydrase were embedded in a polyurethane foam matrix to increase the rate of carbon dioxide conversion to carbonic acid [31].

Here, we address the challenge of developing a continuous biomanufacturing process by assessing lipid production in a 3-D printed continuous flow fixed bed bioreactor using the halophilic, biofilm-forming bacterium, *Marinobacter atlanticus*. A 3-D printed fixed bed bioreactor design was employed here because: (i) it minimizes variability between replicate reactors, enabling higher reproducibility, (ii) the fixed bed bioreactor can be printed as a monolithic structure, eliminating the number of parts necessary to construct the reactor, and (iii) it enables rapid iteration and testing of different reactor parameters (surface area, porosity, reactor volume, etc.) during process optimization. We chose to test *M. atlanticus* as a production strain in a continuous lipid biomanufacturing process because it is known to accumulate lipids in the form of wax esters in response to nutrient limitations, has an established genetic system [32], and is known to degrade an array of non-sugar based carbon sources, including simple organic acids (*e.g.*, succinate) and hydrocarbons [10]. Here, we show that a *M. atlanticus* biofilm is able to produce wax esters in a 3-D printed fixed bed reactor using succinate as the carbon source. We find that removing nitrogen from the seawater medium being pumped into the reactor induces the production of intracellular wax esters in the biofilm cells to a similar concentration as planktonic cells. Further, we characterize the oxygen uptake rate, biomass protein production, and substrate utilization rate of the *M. atlanticus* biofilms under different flow rates. The results obtained here will help to inform future development of a continuous lipid biomanufacturing process based on biofilms formed in a fixed bed bioreactor.

## Methods

### Strain cultivation and medium

*Marinobacter atlanticus* cultures were obtained by streaking freezer stocks onto BB agar plates consisting of, per liter (L): 18.75 g marine broth, 5 g tryptone, 2.5 g yeast extract, 5 g NaCl, and 15 g agar. The plates were incubated at 30 ⁰C for 3 days. Starter cultures were inoculated from colonies in 50 mL plastic centrifuge tubes containing 20 mL low calcium artificial seawater medium with 26 mM sodium succinate (see below). The starter cultures were grown overnight in a 30 °C incubator while shaking at 230 rpm.

Artificial seawater (ASW) medium contains, per L: 27.5 g NaCl, 3.8 g MgCl_2_‧6H_2_O, 6.78 g MgSO_4_‧7H_2_O, 0.7 g KCl, 0.62 g NaHCO_3_, 2.79 g CaCl_2_‧2H_2_O, 1 g NH_4_Cl, 0.05 g K_2_HPO_4_, and 1 mL Wolfe’s Trace Mineral Solution. All components were obtained from Sigma-Aldrich. After autoclaving, the medium was sparged with sterile-filtered (0.22 µm filter, VWR, part #76479-024) CO_2_ until a pH between 6.6 and 6.8 was achieved. Variations to typical ASW medium include: low calcium ASW (0.066 g/L CaCl_2_‧2H_2_O; to favor planktonic growth), low nitrogen ASW (0.1 g/L NH_4_Cl [1.8 mM]; to promote wax ester production), no nitrogen ASW (0 g/L NH_4_Cl; to promote wax ester production in fixed bed bioreactors) and the addition of 5 mM (reactor medium) or 26 mM (flask culture medium) sodium succinate as a carbon source. A succinate concentration of 5 mM was used in the continuous flow fixed bed bioreactor experiments to ensure carbon was not limiting.

### Fixed bed bioreactor design

**Fig. 1:**
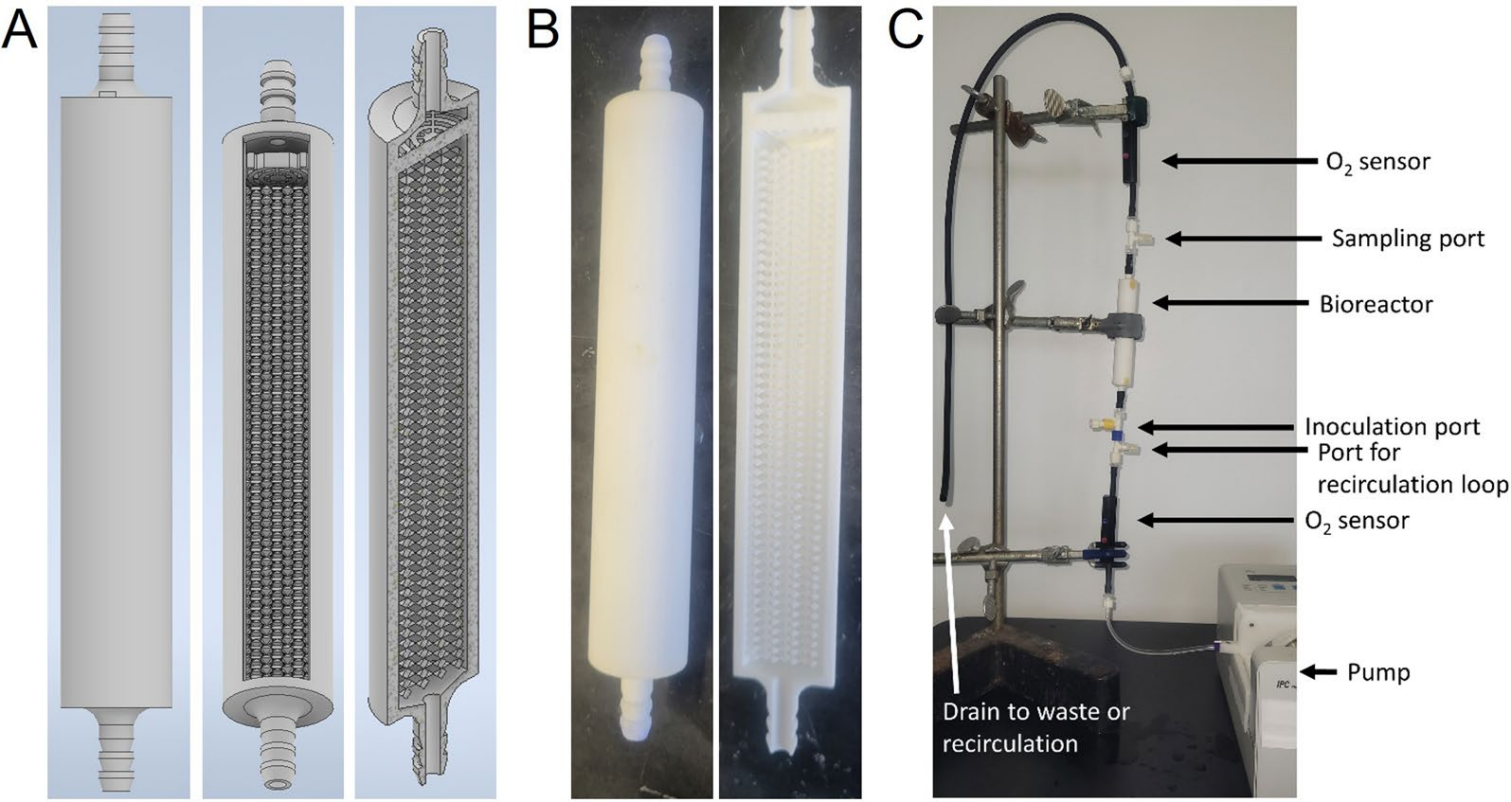
Images of the 3-D printed fixed bed bioreactors and experimental set up used for these experiments. **A** CAD drawings of the reactor showing (left to right) the design used for printing, the reactor with a cutaway showing the fittings, support matrix size, and the dispersion plate. The total height is 11 cm. **B** The printed fixed bed reactors made from nylon. **C** The experimental setup used for experiments, including the influent and effluent oxygen sensors, the inoculation and sample ports, and the pump

Fixed bed bioreactors were printed out of nylon with an EOS P396 selective laser sintering system. The bioreactors used in all experiments were designed in Autodesk Inventor (version 2021.2.1) and are cylindrical with an outer diameter of 1.5 cm and a length of 15 cm (Fig. 1). The main body of the bioreactor was 11 cm tall and a 2 cm barb connection (outer diameter 0.64 cm) was printed on each end to connect inlet and outlet tubing. The internal support matrix was composed of octahedral beads, which consisted of a square at the midplane and 8 equilateral triangles that formed two 4-sided pyramids that protruded in opposite directions from the midplane. Each octahedron had a total height of 2 mm and the length of each side of the midplane was also 2 mm. The octahedra were aligned at the vertices and stacked in the x and y direction to fit as many as possible inside of the cylindrical reactor. This resulted in partial octahedra being printed at the reactor walls. To ensure rigidity of the support matrix, the vertices of neighboring octahedra were overlapped by 5% (0.1 mm). This enabled printing of a single, monolithic fixed bed reactor that can be operated under conditions that could cause washout of packing material in a traditional packed bed reactor.

In addition to a 3-D printed support matrix, a dispersion plate was printed at the reactor inlet to help evenly distribute the flow of medium throughout the reactor. The dispersion plate consisted of a 3 mm diameter circle in the middle of the plate surrounded by alternating 1 mm gaps and 1 mm rings. The dispersion plate was held together and supported within the column by eight rectangular support structures (0.25 mm thickness) that passed through all of the concentric rings and attached to the side of the reactor wall. The total void volume of the reactor was 10 mL.

### Continuous-flow fixed bed bioreactor setup and operation

Prior to each experiment, the bioreactor, tubing, and ports were sterilized. Tubing (1/8″ inner diameter, 1/4″ outer diameter; Norprene, Cole-Parmer, USA) was sterilized via autoclave (121 °C at 15 psi for a minimum of 15 min) and connected to the inlet and outlet of the bioreactor. The inlet tubing was then connected to a peristaltic pump (New Era Pump Systems, Inc., model #9000 or Ismatec, model #C.P.78001-12). The outlet tubing was placed into a waste collection container. Inoculation ports, sampling ports, and oxygen (O_2_) sensors (Pyroscience TOFTC-2 with a Pyroscience FSPRO-4) were inserted into the tubing along the media flow path using barbed Luer fittings upstream and downstream of the bioreactor (Fig. 1). Influent dissolved oxygen concentration was monitored to ensure that oxygenated medium was reaching the reactor inlet. Effluent dissolved oxygen concentration was also monitored. The entire setup was then sterilized by recirculating 70%

ethanol or 10% bleach through the tubing for 20 min using the peristaltic pump. Bleach sterilization was preferred for the recirculation experiments because residual ethanol could be used as an unintended carbon source in the system. For the inoculated single-pass experiments (i.e. medium flows once through the reactor and is not recirculated), either method resulted in the expected performance. Next, sterile MilliQ water was recirculated through the system for an additional 20 min. The reactor was then drained by disconnecting the tubing from the bottom. Prior to inoculation, a media bottle containing sterile low nitrogen ASW containing 5 mM sodium succinate was connected to the inlet of the peristaltic pump and a 18G needle (BD, part #305185) with a 0.22 µm filter (VWR, part #76479-024) was inserted through the top of the medium bottle to allow air exchange. The medium was stirred on a stir plate (IKA Works Inc., model: squid color) at 200 rpm. Next, sterile low nitrogen ASW (with 5 mM sodium succinate) was flushed through the system to waste until a stable signal intensity from the oxygen sensors was obtained. At this point, the oxygen sensors were calibrated to 100% dissolved oxygen concentration and the data logging was initiated.

To prepare the reactor inoculum, the optical density (OD_600 nm_) of the 20 mL overnight culture was first measured. The average OD_600nm_ of the overnight cultures used across all experiments was 0.39 ± 0.05. The remaining culture (ca. 19 mL) was centrifuged at 5250 × g for 30 min (Beckman Coulter, Allegra X-15R). The supernatant was discarded and the pellet re-suspended in 10 mL of sterile low nitrogen ASW (with 5 mM sodium succinate). The tubing just below the inoculation port at the bottom of the bioreactor was removed and the end of the tubing attached to the bioreactor was capped with a sterile cap. This was done to prevent cells from coming into contact with the inlet oxygen sensor. A 10 mL syringe and 23G sterile needle (BD, part #305193) were used to inoculate the bioreactor with 10 mL culture, slowly, over 30 s to avoid shearing the cells. The inoculum was left in the bioreactor for 10 min under static conditions to allow for cell attachment to the support matrix. Next, the bioreactor was reconnected to the inlet tubing and the flow of the medium was restarted at the desired condition (single-pass flow at 0.7 or 4 mL/min; recirculating flow at 0.3 or 3.0 mL/min). A flow rate of 0.7 mL/min was initially chosen to provide enough succinate to not be limiting in the experiments. Oxygen concentration upstream and downstream of the bioreactor was recorded every ten seconds for each experiment. Samples were removed from the sampling port periodically for high performance liquid chromatography (HPLC) analysis. Temperature was not controlled for these experiments and averaged ca. 22 °C.

### Nitrogen cycling experimental conditions

Nitrogen cycling experiments were performed by first operating reactors for 96 h, as described above (carbon and nitrogen non-limiting conditions). After this incubation period, the ASW was switched from low nitrogen (1.8 mM NH_4_^+^) to no nitrogen (0 mM NH_4_^+^) medium. The reactors were then operated for 12 h with flow of carbon-rich and nitrogen-limited ASW, after which the medium was switched back to carbon-rich and nitrogen-rich ASW for 6 h. Therefore, one total cycle length was 18 h with a ratio of nitrogen-rich to nitrogen-limited conditions of 1:2, which was chosen based on previous work [33]. Ten complete cycles of alternating conditions were imposed on the reactor before characterizing the biofilm for lipid production.

To characterize the time to fully replace the reactor medium at 0.7 mL/min flow rate, ammonium was measured at the outlet of the reactor every 15 min after the medium was switched between nitrogen rich and nitrogen poor ASW (and vice versa) using a commercial ammonium test kit (RedSea, Inc.). An abiotic reactor was used for this experiment so that cellular utilization did not influence measurements.

### Wax ester determination

Wax ester concentration was measured at the end of each nitrogen cycling experiment by first draining the loosely attached biofilm cells and associated medium (described as the loosely bound fraction below; ca. 10 mL) contained within the bioreactor into a sterile centrifuge tube as the bioreactor was disconnected from the system. Both the loosely bound fraction and bioreactor were then frozen at -80 °C and lyophilized. The loosely bound fraction was not analyzed for the single-pass and recirculating continuous flow experiments nor were these bioreactors lyophilized or sonicated before hexane extraction. To extract lipids from the biofilms, first a short piece of tubing (ca. 1 inch) was connected to the bottom inlet of the bioreactor and clamped shut. The bioreactor was then filled with hexane and left to sit for 1 h, briefly sonicating (ca. 30 s duration) in a bath sonicator every 20 min. The hexanes (ca. 10 mL) from the bioreactor were then drained into a 20 mL scintillation vial. Similarly, the lyophilized liquid fraction taken from the nitrogen cycling experiments was extracted in ca. 10 mL of hexane and transferred to a separate scintillation vial. Scintillation vials with extracts from the bioreactors and loosely bound fractions were then evaporated to dryness at 50 °C on a centrifugal evaporator (Centrifan, kDscientific). Next, 2 mL hexane were added to the scintillation vials and swirled to re-suspend the wax esters. One milliliter was transferred to a GC–MS vial with screw cap. Octacosane in hexane (10 mg/mL) was used as an internal standard for GC–MS analysis and was added at a concentration of 10 µL/mL (0.25 mM) to each sample. The vials were wrapped with parafilm and stored at − 20 °C until analysis by GC–MS.

### Gas chromatography–mass spectrometry for wax ester analysis

Wax ester samples were analyzed via gas chromatography-mass spectrometry (GC–MS) and gas chromatography (GC) with a flame ionization detector (FID) and autosampler (GCMS-QP2020 NX, GC-2030, AOC-20 s Plus, AOC-20i Plus; Shimadzu Scientific Instruments) according to a modified program from Barney et al*.* [14]. The FID was used to quantify wax esters due to its higher sensitivity compared to the MS detector. The GC–MS contained a fused silica SH-Rxi-5Sil MS column (length: 30 m; inner diameter: 0.25 mm; film thickness: 0.25 µm; Shimadzu Scientific Instruments). The temperature program featured a hold of 1 min at 60 °C, followed by a ramp up to 300 °C at a rate of 10 °C/min, with a final hold period of 15 min at 300 °C. The injector temperature was set to 200 °C and all samples were analyzed with a split of 35. The carrier gas was helium with a linear velocity of 35 cm/s. For the MS, the ion source temperature was 200 °C and the interface temperature was 250 °C. MS data were acquired in scan mode ranging from 50 to 600 m/z. Peaks were identified via MS using GCMS Solution software version 4.52 and the Wiley Registry 12th Edition/NIST2020 library. Peaks were quantified using the GC-FID data in LabSolutions version 5.101. Details of the GC-FID standards used and calibration procedures can be found in the Supplementary Information.

### Biofilm characterization

Oxygen uptake rate (OUR), substrate utilization rate (SUR), biomass concentration, and/or wax ester concentration was measured for each reactor over the duration of the 100 h experiments under continuous single-pass or recirculation flow. OUR was measured once per day for triplicate reactors grown under each of the four different reactor conditions tested (single-pass flow at 0.7 or 4 mL/min; recirculating flow at 0.3 or 3.0 mL/min). First, the peristaltic pump was stopped. Next, the tubing at the top and bottom of the reactor was disconnected and the bioreactor was drained. The bottom tubing was reconnected to the reactor. A fixed fiber oxygen mini sensor probe (Pyroscience, part# OXF1100) was calibrated at 100% in fresh low nitrogen ASW, sterilized in 70% ethanol, then rinsed in sterile low nitrogen ASW (with no carbon source) for 30 s. The dissolved oxygen probe was then inserted into the top of the bioreactor. Using a sterile syringe and needle, 11 mL of low nitrogen ASW with 5 mM sodium succinate was slowly injected into the inoculation port below the bioreactor. The oxygen concentration was monitored for 20 min, after which the dissolved oxygen probe was removed and rinsed with 70% ethanol and sterile low nitrogen ASW (no carbon). The tubing at the top of the bioreactor was reconnected, and the peristaltic pump was restarted. The OUR was calculated by measuring the decrease in oxygen over time (until % O_2_ decreased below 5%). Percent dissolved oxygen in the reactor was converted to mmol O_2_ based on standard tables of oxygen solubility in 35 ppt salt water at 25 °C and atmospheric pressure. OUR (mmol O_2_/L/h) was obtained by taking the slope of a linear regression through the oxygen concentration vs. time curve.

Substrate utilization rate was determined by operating triplicate bioreactors with continuous recirculation of the medium and no fresh medium addition. Samples were taken daily for HPLC analysis to characterize the decrease in succinate over time. A linear regression was used to calculate the substrate utilization rate from these datasets.

To estimate biomass, following extraction via hexane, triplicate bioreactors were filled with 0.2 N NaOH (ca. 10 mL) and left to sit for 1 h. Each bioreactor was then drained into a 50 mL centrifuge tube. Next, the bioreactors were filled with sterile water (ca. 10 mL) and this was emptied into the same 50 mL centrifuge tube for that bioreactor. The tubes were then stored at − 20 °C. To analyze total protein, samples were subjected to a freeze/thaw procedure (thaw at 90 °C for 30 min, freeze at − 20 °C for 1 h, repeat two more times, thaw at room temperature (ca. 23 °C) for 1.5 h) prior to performing a protein concentration assay (Invitrogen Qubit protein assay, part #Q33211).

## Results

### Nitrogen cycling induces lipid production in M. atlanticus biofilms

**Fig. 2:**
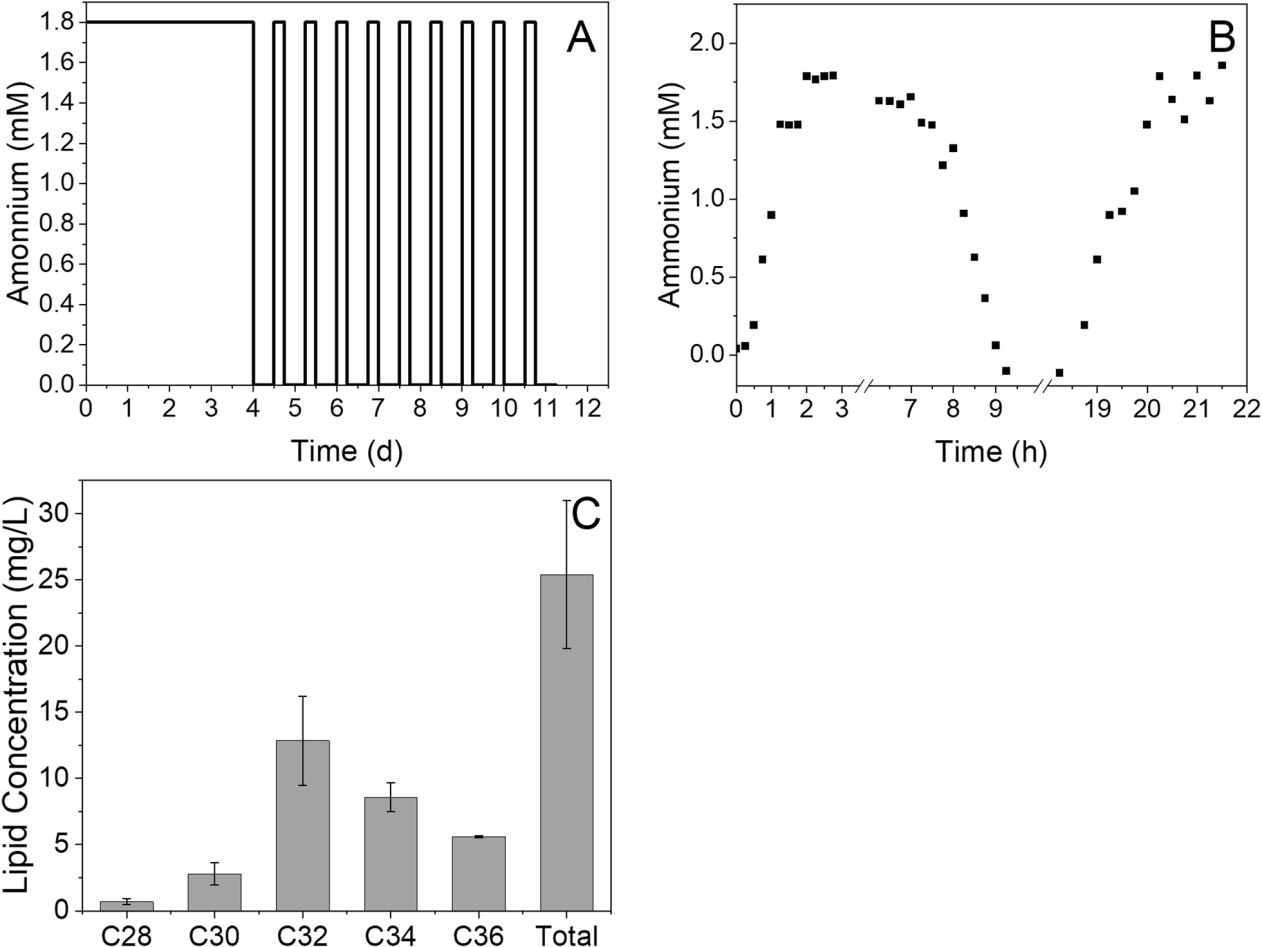
**A** The ammonium concentration in the medium supplied to the bioreactor over time during growth (Day 1–4) and nitrogen cycling (Day 4–11). **B** The ammonium concentration at the reactor outlet during three transitions between nitrogen-rich and -limited conditions. This experiment was performed under abiotic conditions so microbial activity would not affect the results. **C** Total average lipid concentration and profile from *M. atlanticus* biofilms grown in the fixed bed bioreactor after ten total cycles. Error bars represent the standard deviation of three independent replicates

*M. atlanticus* biofilms incubated in fixed bed bioreactors for 96 h on a continuous flow of seawater medium containing succinate (5 mM) and nitrogen (1.8 mM NH4^+^) were subjected to 10 cycles of alternating nitrogen-poor (0 g/L NH4^+^; nitrogen-limited) conditions for 12 h followed by nitrogen-rich conditions (1.8 mM NH4^+^; nitrogen-rich) for 6 h (total of 96 h of growth followed by 174 h of nitrogen cycling; the experiment was terminated at the end of the nitrogen-limited portion of the cycle; Fig. 2A).

Monitoring the ammonium concentration at the reactor outlet indicated that 2 h of pumping at 0.7 mL/min was required to fully exchange the medium in the reactor under abiotic conditions (Fig. 2B), significantly shorter than the duration of the nitrogen-rich or nitrogen-limited portion of the cycle. The average wax ester concentration extracted from the reactors after the nitrogen cycling experiments was 26 ± 5 mg/L, _reactor volume_ (i.e., mass of total wax esters normalized to reactor volume; Fig. 2C). The tightly attached biofilm in the bioreactor contained approximately 60% of the wax esters recovered and the loosely bound fraction that drained out of the bioreactor at the end of the experiment contained 40% of the total. We did not attempt to extract wax esters from the medium that exited the reactor through the outlet during the course of the experiment. The extracted wax ester profile was dominated by multiple compounds with a carbon chain length of 32 (C_32_; 50 ± 3%), followed by C_34_ compounds (34 ± 3%) and C_30_ compounds (11 ± 1%). Wax esters with carbon chain lengths of C_28_ (3 ± 0.3%) and C_36_ (2 ± 0.1%) were a minor proportion of the total.

*M. atlanticus* biofilms that were characterized immediately after the 100 h incubation period and not subjected to nitrogen cycling did not produce detectable amounts of wax esters under the extraction conditions used here regardless of the flow rate or whether the medium was recirculated.

### Oxygen availability is growth-limiting in M. atlanticus biofilms

**Fig. 3:**
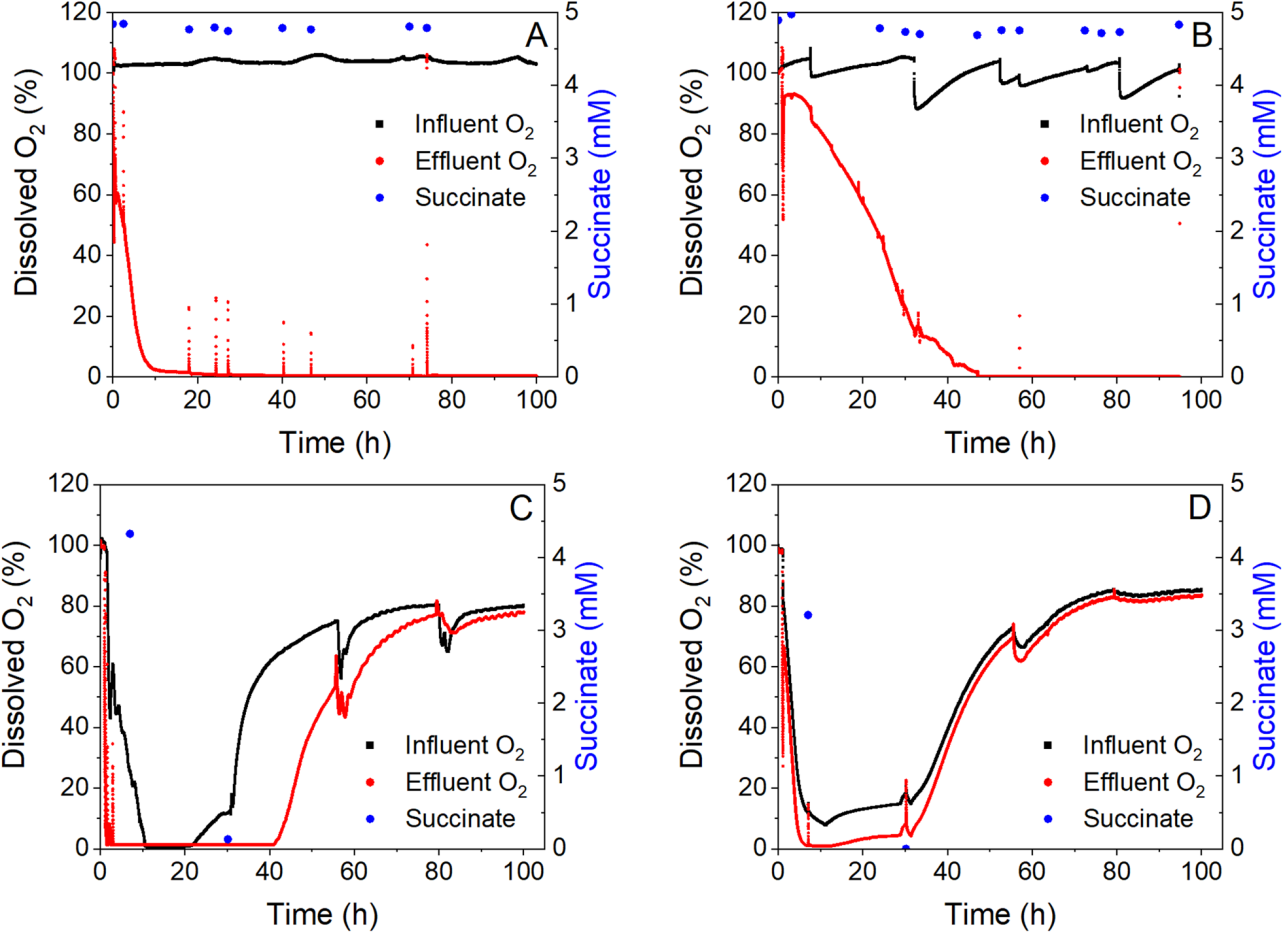
Influent (black line) and effluent (red line) dissolved oxygen (O_2_) and effluent succinate concentration (blue circles) measured from fixed bed bioreactors over the course of an experiment under **A** single pass flow through at 0.7 mL/min flow rate, **B** single pass flow through at 4 mL/min flow rate, **C** recirculation of reactor medium at 0.3 mL/min and **D** recirculation of reactor medium at 3 mL/min. Data presented here are representative of triplicate reactors. Replicates are shown in Fig. S1

Reactor effluent oxygen concentration was monitored for all reactors tested as an indicator for respiration of the biofilm due to succinate oxidation. In abiotic experiments, influent and effluent dissolved oxygen remained at 100% saturation for the duration of the experiment. We found that the effluent oxygen was completely consumed (< 2% saturation) in the reactors colonized by *M. atlanticus* biofilms over time, regardless of the flow rate used or whether medium was recirculated or had a single pass through the reactor. However, the time to oxygen depletion was dependent on the operating conditions of the reactor. When medium was fed continuously through the reactor at 0.7 mL/min with no recirculation, dissolved oxygen was not detected in the effluent after 9.7 ± 4.4 h (Fig. 3A). When the flow rate was increased to 4 mL/min oxygen was detected until between 26 and 68 h (46.6 ± 21 h; Fig. 3B).

To further assess the rate of oxygen consumption in the bioreactor and any oxygen intrusion in the recirculation loop, reactors were also tested under recirculating flow conditions with no addition of new medium. When medium was continuously recirculated at 0.3 mL/min (hydraulic residence time [HRT] in the reactor 33 min), oxygen was no longer detectable after 1.2 ± 0.2 h (Fig. 3C). By increasing the recirculation rate 10 × to 3 mL/min (HRT 3.3 min), the oxygen was consumed in 4.6 ± 1 h (Fig. 3D).

**Fig. 4:**
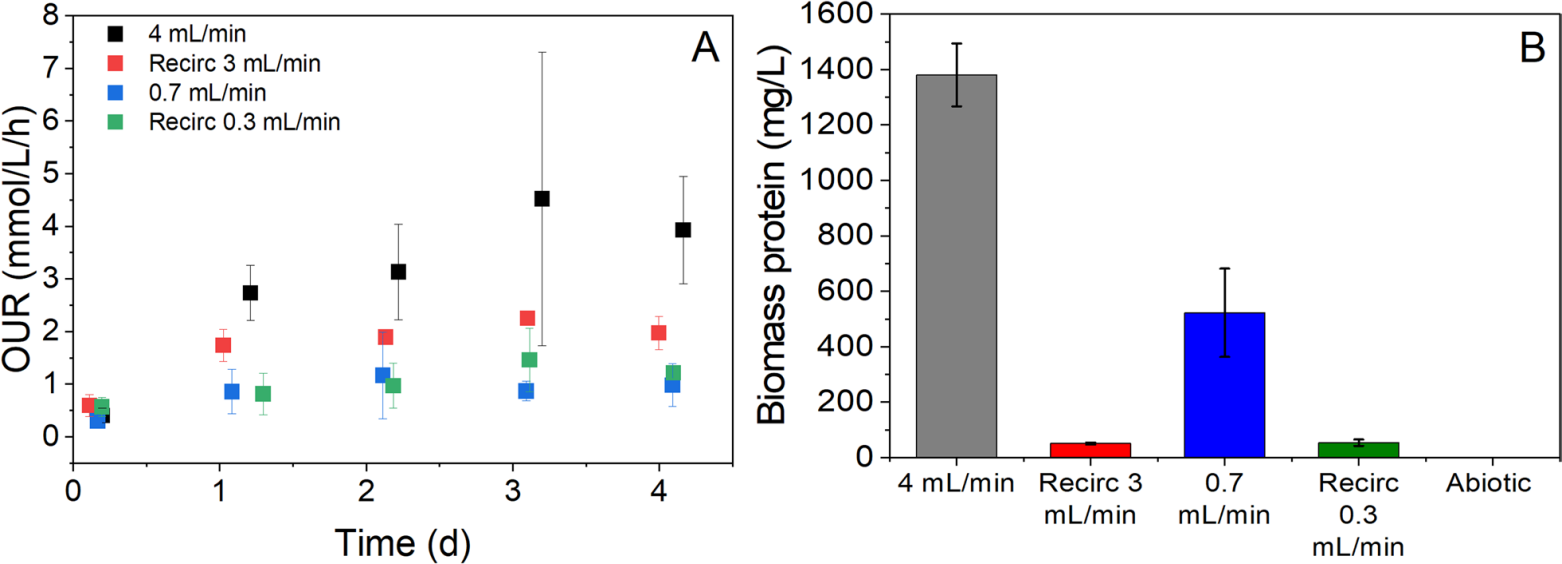
**A** Oxygen uptake rate of *M. atlanticus* biofilms when grown under different operating conditions, *i.e.* different flow rates and with or without medium recirculation. **B** Biomass produced over the course of the 100 h incubation under different flow rates and conditions, as measured by extracted protein. Data are the average of three biological replicates. Legend: 4 mL/min and 0.7 mL/min denote continuous single-pass operation of the reactors. Recirc 3 mL/min and Recirc 0.3 mL/min denote reactor operation under continuous recirculation of medium at the specified rate. Error bars represent the standard deviation of three independent replicates

In order to directly compare respiration rates of biofilms established under different reactor operational conditions, we performed daily oxygen uptake rate (OUR) measurements, whereby an external oxygen sensor was used to measure the rate at which the biofilms grown under different operating conditions depleted oxygen from a 10 mL bolus of fully oxygenated seawater medium with 5 mM succinate (Fig. 4A). Growing biofilms in the bioreactors under a continuous, single-pass flow rate of 4 mL/min resulted in formation of a biofilm with a significantly higher OUR (4 mmol O_2_/L/h) than the other operating conditions tested here. The biofilms grown with a recirculation rate of 3 mL/min had an OUR of ca. 2 mmol O_2_/L/h, followed by the 0.3 mL/min recirculation and the 0.7 mL/min single pass flows both with an OUR of ca. 1 mmol O_2_/L/h.

For both the 0.7 mL/min and 4 mL/min flow rate experiments (Fig. 3A, B), influent dissolved oxygen remained near 100%, indicating that fully oxygenated ASW medium was being pumped from the medium reservoir into the bioreactor for the duration of the experiment. Under recirculating conditions (Fig. 3C, D), the oxygen in the medium was consumed to below 2% and then steadily increased to ca. 80% of saturation, likely due to oxygen intrusion into the recirculation line after the carbon source was consumed.

Biomass production in the fixed bed reactors was used as an indicator for oxygen availability to the biofilm (Fig. 4B). To assess biomass growth in reactors operated under different conditions, cell protein was quantified at the end of each experiment (i.e., at 100 h). Operating the reactors at a flow rate of 4 mL/min resulted in a significantly (t-test, *p* < 0.05) higher protein concentration (1380 ± 110 mg protein/L) extracted from the biofilm than any of the other conditions tested. The protein content of the biofilm grown under a flow rate of 0.7 mL/min (520 ± 160 mg protein/L) resulted in approximately half of the protein concentration in the reactor. The reactors operated with recirculation and no addition of fresh medium produced similar amounts of biomass protein, which was significantly (t-test, *p* < 0.05) less than the other conditions (51 ± 4 mg/L under 3 mL/min recirculation and 54 ± 12 mg/L under 0.3 mL/min recirculation).

### Biofilm substrate utilization rate

Succinate concentration remained constant during single pass continuous experiments (i.e., without medium recirculation, Fig. 2), indicating that substrate utilization rate was far below the rate at which succinate was added to the reactor. During recirculation tests, we observed a concurrent decrease in the succinate concentration with decreasing dissolved oxygen (Fig. 3C, D). A subsequent increase in dissolved oxygen, likely due to intrusion in the recirculation line, coincided with a decrease in succinate concentration below the HPLC detection limit, indicating that oxygen and succinate removal were the result of microbial activity. From the linear decrease in succinate concentration over time, we were able to determine the substrate utilization rate of the *M. atlanticus* biofilms exposed to medium recirculation at a rate of 3 mL/min (0.20 ± 0.12 mmol succinate/L/h) or 0.3 mL/min (0.16 ± 0.03 mmol succinate/L/h). The larger standard deviation for the 3 mL/min condition is due to one replicate that consumed succinate significantly more quickly than the others, the cause of which was not apparent from the data collected.

## Discussion

The results presented here demonstrate that wild-type *M. atlanticus* biofilms grown in a continuously operated fixed bed bioreactor produce wax esters at a similar titer (25 mg/L) as planktonic cultures grown in shake flasks without any optimization of the bioreactor process. The lipid concentration produced by *M. atlanticus biofilms* is within the range observed for other planktonic lipid accumulating organisms, which spans a wide range from ca. 10 to 150 mg/L (Table 1).

**Table 1:** Wax ester production by different microorganisms

Microorganism	Carbon source	Cell state	Wax esters produced (mg/L)	References
*Alcanivorax borkumensis SK2*	Pyruvate	Planktonic	0	[34]
*Alcanivorax jadensis T9*	Pyruvate	Planktonic	17	[34]
*Marinobacter hydrocarbonoclasticus* SP17	Pyruvate	Planktonic	145	[34]
*Thalassolituus oleivorans* MIL-1	Pyruvate	Planktonic	0	[34]
*Acinetobacter baylyi* ADP1	Glucose	Planktonic	8	[35]
*Marinobacter atlanticus*	Succinate	Planktonic	25	[32]
*Marinobacter atlanticus*	Succinate	Biofilm	26	This study

In one previous study [34], lipid production was characterized from multiple organisms grown on pyruvate, another simple organic acid substrate. The results showed that *Marinobacter hydrocarbonoclasticus* SP17 produced ca. 145 mg/L of wax esters, at the high end of the range of reported values. Other organisms, such as *Alcanivorax jadensis* T9, produced similar (ca. 10 mg/L) amounts of wax esters as *M. atlanticus* (25 mg/L; this study and [32]). Another wax ester accumulating strain, *Acinetobacter baylyi* ADP-1, was observed to produce approximately 8 mg/L of wax esters when grown on high concentrations (110 mM) of glucose [35]. In all of these previous studies, nitrogen was depleted in the culture medium before wax ester analysis took place.

Removal of ammonium from the reactor medium in nitrogen cycling experiments appears to be key in the production of wax esters, indicating that, as with planktonic cells [36], nitrogen limitation plays a critical role in the activation of intracellular lipid production pathways. When the concentrations of succinate and ammonium were kept constant (5 mM succinate with 1.8 mM NH_4_^+^) in the reactor medium, we did not observe detectable wax ester production, supporting the notion that lipid production in *M. atlanticus* biofilms is induced by nitrogen depletion. Medium with the same concentration of ammonium was used previously to make nitrogen-limited ASW to induce wax ester production in batch cultures of planktonic *M. atlanticus* cells [32]. Under batch conditions in a shake flask, ammonium (and succinate) is continuously depleted in these experiments resulting in conditions under which we expect lipid production. Here, we imposed ten cycles on the biofilms to ensure that the biofilms were exposed to nitrogen limiting conditions under a continuous flow of medium to favor conditions for wax ester production. To date, we have not characterized the amount of time that biofilms need to be exposed to nitrogen-limited conditions to accumulate a detectable amount of lipids. The effect of nitrogen cycling was only characterized at 0.7 mL/min flow rate due to limitations with operating our current setup at a higher flow rate for an extended period. We note that operating the reactor at 4 mL/min resulted in an approximately 3 × increase in biomass content in the reactor, indicating that higher flow rates and/or oxygen concentrations would lead to more cells available for lipid production in the biofilm reactor and could result in significantly higher lipid titers.

As biofilm-based biomanufacturing processes are further developed, it is critical to evaluate the parameters underpinning the performance metrics observed to aid in identifying limitations toward developing an economically viable process. In these un-optimized experiments, we observed similar lipid production after more than double the run time (275 vs 100 h) of the batch tests, a promising result for continuous lipid production. However, the bioreactor-based production experiments here used 11.5 L of medium, which far exceeds the medium used (1 L) in the batch reactors previously tested. Optimization of the process, including medium recirculation and methods to continuously assess lipid production over time could have a significant effect on the resources required to obtain the desired production rate from the biofilm-based process. As previously shown, continuous production minimizes process downtime [22] and leads to greatly enhanced process economics [21]. Here, we have also begun to establish baseline process parameters, such as OUR and SUR, for biofilm-based manufacturing processes, which are necessary to understand and further develop these biofilm-based processes toward implementation at larger scales. One significant challenge to further development of this process is the harvesting of a lipid material that is produced intracellularly. A few potential solutions to overcome this hurdle are to: (i) implement a bacterial “milking” process were a bolus of solvent is used to periodically extract the lipids from the biofilm [37–39]; (ii) to recover the lipids from the loosely bound fraction of the biofilm periodically; or (iii) to utilize a production host with a natural or engineered capability to export accumulated lipids [40]. In our estimation, each of these paths is a worthwhile pursuit and will be the focus of future efforts.

Data presented here indicate that oxygen availability was a critical factor for the *M. atlanticus* biofilms. As can be seen in all experiments, the oxygen concentration in the medium decreased from saturation to zero between the reactor inlet and outlet, while there was no detectable change in the succinate concentration, indicating that oxygen was the limiting reactant in the system. The maximum OUR observed here (4 mmol O_2_/L/h; 0.035 mg O_2_/L/s) is similar to previous reports on mixed community heterotrophic biofilms grown on trickling filters in a wastewater treatment plant [41] and on simulated wastewater in a gravity sewer [42]. An increase in the medium flow rate from 0.7 to 4 mL/min (5 × higher rate of oxygen delivery into the reactor), resulted in a biofilm that could consume oxygen at a rate 4 × higher than other biofilms tested. In addition, there was a three-fold increase in the biomass protein at the higher medium flow rate. Taken together, these results indicate that the biofilm is limited for growth by the amount of oxygen supplied to the system. When biofilms were grown under recirculating conditions, they exhibited a similar oxygen uptake rate to the 0.7 mL/min single-pass reactors despite producing approximately 5 × less biomass. We ascribe this result to formation of a thicker biofilm in the single-pass flow reactors where the oxygen is consumed before it fully penetrates into the biofilm. As can be seen in the experimental data (Fig. 2C, D), there is a large increase in the oxygen concentration at the outlet of the reactor after the succinate is fully consumed, likely due to oxygen leaks in the tubing connections. This would enable a continuous, but limited, supply of oxygen into the reactor without continuous addition of oxygenated medium. We did not extract the biomass in the recirculation line, so it is possible there were active cells in the system that were not included in the biomass measurements. Reactor oxygen concentration has been implicated as a determining factor for lipid accumulation in another marine bacterium, *Alcanivorax borkumensis* [40]. In this previous study, the concentration of lipids produced decreased as the concentration of oxygen decreased below saturation. The type of lipid accumulated also differed based on the oxygen delivery strategy. However, we did not observe wax ester production in the reactors within the initial 100 h incubation period despite an oxygen gradient from saturated to completely consumed across the length of the reactor under different flow conditions. This could be due to the limit of detection of our instruments if only a relatively small proportion of the biofilm was exposed to an oxygen-limited environment that may be conducive for lipid accumulation. The effect of oxygen on the production of wax esters will be studied more rigorously in the future because of its importance in the system.

Succinate consumption was not observed during single pass continuous operation due to the high rate of supply compared to the consumption rate. Given that the working volume of the bioreactor used here is approximately 10 mL, succinate is supplied to the reactor at a rate of 3.6 µmol/min. The succinate utilization rate was observed to be 0.20 ± 0.12 mmol/L/h under 3 mL/min recirculating conditions, indicating that the biofilm was consuming 0.03 µmol/min in the reactor, 100 × less than the rate of supply. We note that the SUR reported here likely underestimates the consumption rate of a biofilm grown under single-pass conditions given that significantly more biomass was produced in reactors operated under those conditions. However, the succinate concentration did not change over the length of the column for reactors with operating conditions that led to higher biomass either, indicating that the SUR estimated here is reasonable.

## Conclusions

The data presented here show that biofilms of the marine bacterium, *Marinobacter atlanticus*, are capable of accumulating wax esters when grown in a fixed bed bioreactor at a similar concentration to what has been observed from planktonic cells grown in shake flasks. The production of lipids by the biofilm is controlled by exposure to a nitrogen poor environment in the reactor. Further, oxygen availability was shown to have a significant impact on the production of biomass within the reactor, which ultimately affects the number of cells available to form the lipid products. The results presented here indicate the potential for lipids to be produced continuously from a naturally formed biofilm in a fixed bed bioreactor. Further, we established baseline OUR and SUR for *M. atlanticus* biofilms. Continued optimization of the fixed bed bioreactor process should focus on decoupling the addition of oxygen from the addition of liquid feed, optimal carbon concentration in the reactor, determining the optimal C:N ratio, recirculation rate of reactor medium, and the optimal ratio of nitrogen-rich to nitrogen-limited periods during a cycle.

## Supplementary Information

Supplementary Material 1.

## Data Availability

The authors declare that the data supporting the findings of this study are available within the paper and its Supplementary Information files. Should any raw data files be needed in another format they are available from the corresponding author upon reasonable request.
