# Supplementary material for: Lipid production from biofilms of Marinobacter atlanticus in a fixed bed bioreactor
Source: Microb Cell Fact. 2024 Dec 19;23:336. doi: 10.1186/s12934-024-02617-5 (PMC11657173; doi:10.1186/s12934-024-02617-5)
Supplement: Supplementary file 1 — Supplementary Material 1. [file 12934_2024_2617_MOESM1_ESM.docx]

Supplementary Information for:

**Lipid production from biofilms of *Marinobacter atlanticus* in a fixed bed bioreactor**

Matthew D. Yates^1*^, Rebecca L. Mickol^1^, Joseph S. Tolsma^1||^, Maryssa Beasley^1^, Jamia Shepard^1^, and Sarah M. Glaven^1^

^1^Center for Bio/Molecular Science and Engineering, Naval Research Laboratory, Washington, DC, 20375

*Corresponding Author: matthew.d.yates7.civ@us.navy.mil

^||^Present address: Catalent Pharma Solutions, Kansas City, MO, 64137

*GC-FID standards and calibration procedures*

One set of standards, consisting of 14 separate concentrations spanning a range from 0.6 µM to 8 mM, was prepared in hexane. Stock solutions of individual standards were initially prepared at 15 – 40 mM, depending on the compound. From each of these separate stocks, specific aliquots were combined to obtain a mixture with each of the compounds at molarities between 4 – 8 mM. From this standard mixture, thirteen additional concentrations were created through serial dilution. The standard mix consisted of stearyl stearate (C_36_H_72_O_2_), cetyl palmitate (C_32_H_64_O_2_), stearyl alcohol (C_18_H_30_O), cetyl alcohol (C_16_H_34_O), palmitic acid (C_16_H_32_O_2_), and methyl myristoleate (C_15_H_28_O_2_). To each standard mix, 10 mg/mL octacosane in hexane was added as an internal standard (10 µL/mL; 0.25 mM). All wax ester/standard peak areas were normalized by the peak area for octacosane before further analysis.

To quantify wax esters, calibration curves were created using five of the six standard compounds (palmitic acid was found to be unreliable over the range of concentrations tested). Because a mixture of wax esters with a range of carbon chain lengths were detected in the samples, the quantification was reported in terms of moles carbon, instead of moles of the specific compound. This generated a calibration curve consisting of five compounds, spanning a range in carbon number (C15 – C36). Wax ester mass (mg) was estimated using the following molecular weights for individual peaks with certain carbon numbers. Molecular weights were also informed from MS identifications: C28, 424.7 g/mol; C30, 452.8 g/mol (cetyl myristate); C32, 476.8 g/mol, 478.8 g/mol, 480.8 g/mol (cetyl palmitate); C34, 504.9 g/mol, 506.9 g/mol, 508.9 g/mol; C36, 537 g/mol. Specific identifications of each individual wax ester peak was outside the scope of this study.

*Determining the GC-FID baseline*

In order to establish a standard baseline for GC-FID, the root mean square (RMS) was calculated from the baselines of multiple blanks (*i.e.*, only hexanes and the internal standard; minimum of four blanks). The data used to calculate the baseline of each blank was taken after the octacosane signal had decayed. The multiple baseline values obtained were averaged and the result was taken as the standard baseline. The limit of detection (LOD) and limit of quantification (LOQ) were then calculated from the baseline using the standard definitions [34, 35]:

LOD = 3 * baseline (1)

LOQ = 10 * baseline (2)


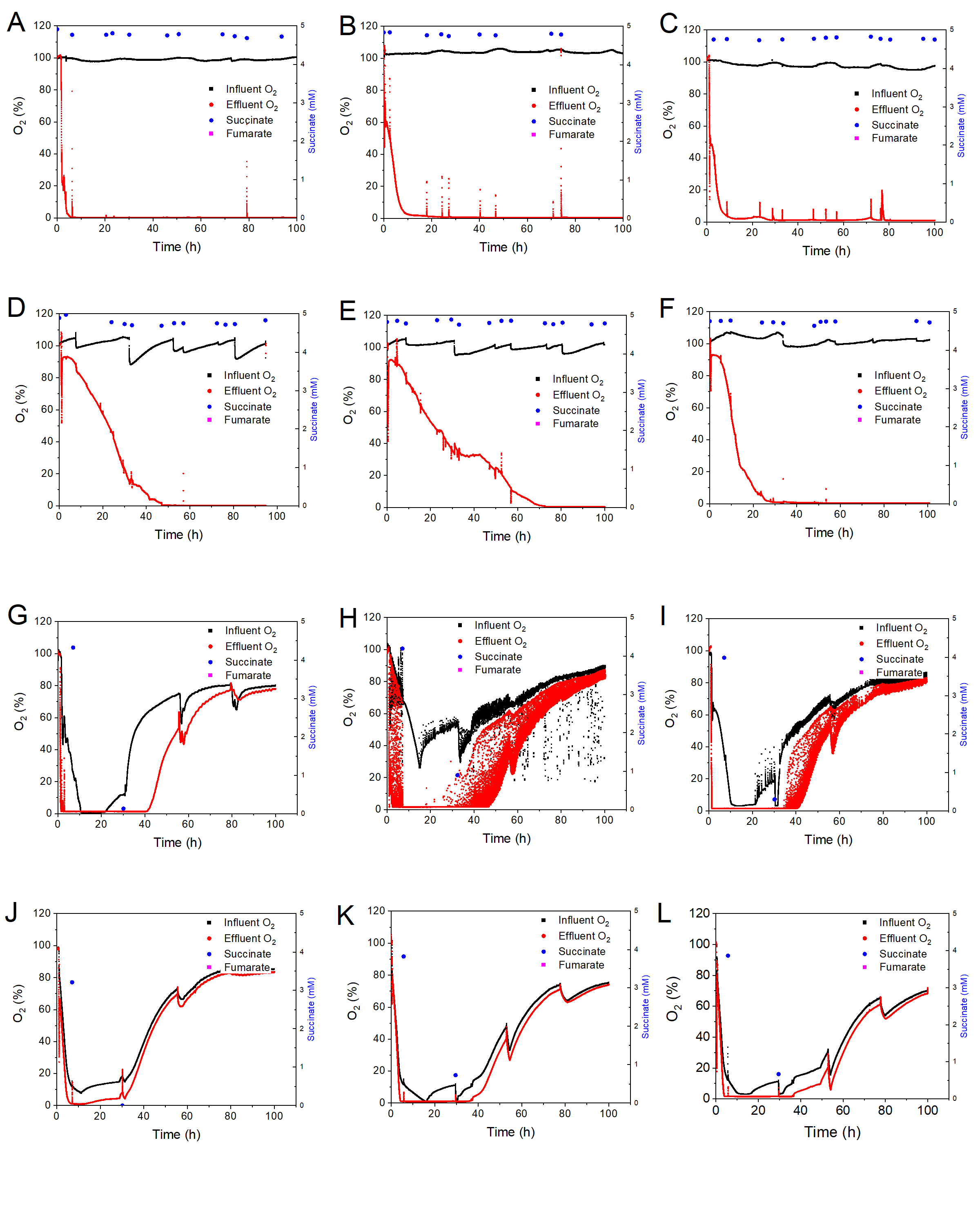


**Figure SI1.** Replicate reactors operated under the following conditions: **(A-C)** single pass flow through at 0.7 mL/min flow rate, **(D-F)** single pass flow through at 4 mL/min flow rate, **(G-I)** recirculation of reactor medium at 0.3 mL/min and **(J-L)** recirculation of reactor medium at 3 mL/min. The spread of the data in Panels H and I were due to issues with the data collection system.
